# Nutritional Status and Symptoms in Preschool Children With Autism Spectrum Disorder: A Two-Center Comparative Study in Chongqing and Hainan Province, China

**DOI:** 10.3389/fped.2020.00469

**Published:** 2020-09-03

**Authors:** Jiang Zhu, Min Guo, Ting Yang, Xi Lai, Ting Tang, Jie Chen, Ling Li, Tingyu Li

**Affiliations:** ^1^Children's Nutrition Research Center, Ministry of Education Key Laboratory of Child Development and Disorders, National Clinical Research Center for Child Health and Disorders, China International Science and Technology Cooperation Base of Child Development and Critical Disorders, Children's Hospital of Chongqing Medical University, Chongqing, China; ^2^Chongqing Key Laboratory of Child Health and Nutrition, Chongqing, China; ^3^Department of Children Rehabilitation, Hainan Women and Children's Medical Center, Hainan, China

**Keywords:** autism spectrum disorders, dietary, nutrition, micronutrients, vitamin, children

## Abstract

**Objective:** The study aimed to compare the nutritional status and symptoms of preschool children with autism spectrum disorder (ASD) from two regions of China, and to analyze the association between nutritional status and symptoms of ASD.

**Methods:** In this cross-sectional study, 738 ASD children and 302 typically developing children (TD) were recruited from Chongqing and Hainan of China. Symptoms of ASD children were evaluated with the Autism Behavior Checklist (ABC), Social Responsiveness Scale (SRS), and Childhood Autism Rating Scale (CARS). Neurodevelopment of ASD children was assessed with the Gesell Developmental Scale (GDS). Nutritional status was evaluated by anthropometric measures, biochemical detection of micronutrients, and providing questionnaire and food frequency questionnaire (FFQ) to caregivers.

**Results:** Comparing ASD children with local TD children, ASD children consumed fewer whole grains, milk and dairy products, beans and soy products, vegetables, and fruits than local TD children in both regions. The serum concentrations of folate, vitamin B12 (VB12), and vitamin D (VD) were consistently lower in ASD children in both regions. Comparing the ASD children between the two regions, the ASD children in Chongqing had significantly higher mean scores of CARS, SRS, and ABC than those in Hainan. The ASD children in Chongqing consumed fewer whole grains, seafood, and fruits than those in Hainan. The serum concentrations of ferritin, vitamin A (VA), VB12, and VD were reduced in the ASD children of Chongqing than those in Hainan, and the ASD children in Chongqing had higher deficiency rates of zinc, ferritin, VA, and VD than those in Hainan. The serum levels of VA, VD, and folate showed a negative association with symptom scores of ASD children. VD and zinc levels had a positive association with the GDS scores of ASD children.

**Conclusions:** ASD children exhibit a higher risk of nutrient deficiencies than neurotypical children, and there are regional differences in the nutritional status of ASD children. Micronutrients VA, VD, folate, and zinc levels were correlated with symptoms and development of ASD children. Therefore, it is essential to provide detailed nutrition evaluation and individualized nutrition interventions for ASD children from different backgrounds.

## Introduction

Autism spectrum disorders (ASDs) are wide-spectrum neurodevelopmental disorders characterized by early appearing social communication deficits and repetitive sensory motor behaviors (1). The prevalence of ASD has been markedly increasing around the world in recent decades. Statistics estimate that prevalence of ASD global was 1% in 2012 (2), and the prevalence in developed countries was estimated to be 1.5% in 2017 (3). Multiple genetic, environmental (including infection, medication, chemicals, and nutrients), and immunologic factors take part in the pathogenesis of ASD (3).

Studies emphasize that children with ASD are nutritionally vulnerable due to their problematic eating behaviors, gastrointestinal symptoms, food allergies, and metabolic abnormalities (4, 5). Micronutrients play indispensable roles in maintaining the structure and regulating the function of the nervous system. An inadequate intake of some micronutrients could have a harmful impact on neurodevelopment (6). Studies reported decreased dietary intake and serum concentrations likely below the reference range or lower than neurotypical controls of folate (7), vitamin B12 (VB12) (7, 8), vitamin D (VD) (9, 10), vitamin A (11, 12), iron (13), and zinc (12) in children with autism. Nevertheless, there are inconsistent findings. Molloy et al. (14) and Basheer et al. (15) found that VD levels were not different between ASD children and those in the neurotypical group, while children in both groups had a high rate of VD deficiency. Ugur (16) found that serum VD and folate levels of ASD children were not significantly different from those of neurotypical controls. Hope et al. (17) found that patients with neurodevelopmental disorders (including ASD) have a significantly higher serum level of VB12 than neurotypical children. Whether ASD children present malnutrition similarly or more frequently than typical children is inconclusive because the nutritional status of an individual is a consequence of complex mechanisms and interactions, which could be affected by ethnicity, diet, and other factors in different countries or regions. The differences in the measurement methods and status also affect the variations in micronutrient concentrations. In our previous studies, we found that ASD children had a higher risk of malnutrition than control children in Chongqing (11, 18). In the present study, we recruited preschool ASD children and typically developing (TD) children from two regions—Chongqing and Hainan province. It is intended to compare the symptoms and the nutritional status of ASD children from two regions and analyze the nutritional factors associated with the autism symptoms. This study may help in the future management of children with ASD.

## Methods

### Study Areas

Chongqing is situated in the southwest of China with latitude of 28″10′-32″13′ N. Hainan province is an island situated in extreme southern China with latitude of 18″10′-20″10′ N (Figure Supplement 1, the locations of Chongqing and Hainan province in China). The economic conditions of the two regions are at a comparable level and reflect the middle-class Chinese population. However, Hainan is richly endowed by nature with pleasant tropical weather and sunlight, and abundant natural resources based on its unique geographical condition. Chongqing is known as the mountain city and fog city with extremely long rainy seasons and a high level of fog most of the year.

### Participants

A total of 738 ASD children aged 2–6 years were recruited for the study, 445 of which were recruited from the Children's Hospital of Chongqing Medical University and special education school of Chongqing, China, and 293 from the Maternal and Child Care Health Hospital of Hainan Province, China. Inclusion criterion was a diagnosis of ASD, which was made by two developmental pediatricians independently through a series of structured interviews according to the Diagnostic and Statistical Manual of Mental Disorders, Fifth Edition (DSM-5) criteria. The Childhood Autism Rating Scale (CARS) (scores for ASD children should be >30) (19) was used as an assistant in diagnosing. Exclusion criteria included a history of other neurodevelopmental disorders, neurological or psychiatric diseases, known genetic disorders, major physical illness, a recent infection, recent use of special diets, and recent supplement of high-dose vitamins or minerals. Symptoms of ASD children were evaluated with the Autism Behavior Checklist (ABC) (19), Childhood Autism Rating Scale (CARS), and Social Responsiveness Scale (SRS) (20). Higher scores of ABC, SRS, and CARS scores represent more serious autistic symptoms. Neurodevelopment in ASD children was assessed with the revised Gesell Developmental Scale (GDS) (21), which is extensively used in China to evaluate cognitional and behavioral development, and the development quotient (DQ) scores reflect the levels of intellectual and behavioral development. DQ <70 indicates developmental delay, and the lower the DQ score, the more severe the developmental delay.

Additionally, 302 residence- and age-matched typically developing (TD) children were randomly recruited from local nursery schools as control groups, including 201 from Chongqing and 101 from Hainan province, China. These participants are healthy, and they did not have any signs of developmental disorders or psychiatric diseases. Other exclusion criteria were the same as for the ASD group.

Participation in this research was voluntary (Figure Supplement 2, participant flow chart). Parents of all children were willing to let their children participate and signed consent forms. The study protocol was approved by the Institutional Review Board of the Children's Hospital, Chongqing Medical University. This clinical study was also registered in the Chinese Clinical Trial Registry (ChiCTR; registration number: ChiCTR-ROC-14005442).

### General Condition Survey and Food Frequency Questionnaire

The caregivers of the ASD and TD children completed a series of questionnaires conducted by in-person interviews between the standardized trained investigators and caregivers. The following data were obtained: the children's general condition (e.g., name, age, gender, ethnicity, family economic status, and parental education level), medical history, eating behavior, gastrointestinal problems, etc. Dietary intake has been assessed by well-trained dieticians using a semiquantitative food frequency questionnaire (FFQ) (22). The original version of the FFQ included 8 food groups and 86 food items that were frequently consumed by the Chinese population, while 11 food groups were adapted into this study. The white meat group was divided into three groups: poultry meats, freshwater fish, and seafood, and the whole grains group was added. For each food item, caregivers were asked to report their children's average consumption frequency over the past month and the estimated portion size and weight.

### Anthropometric Measurements

Anthropometric measures were conducted by certified surveyors with a standardized approach (23). *Z*-scores were calculated for height-for-age (Z_HA_), weight-for-age (Z_WA_), body mass index-for-age (Z_BMIA_), and weight-for-head circumference (Z_HCA_) with World Health Organization (WHO) Anthro and AnthroPlus software (Anthro for Personal Computers, Version 3.01, WHO, 2009), with the use of the 2005 version of the WHO child growth standard for children aged 0–5 years and the 2007 version for children and adolescents aged 5–19 years.

### Detection of the Biochemical Index for Nutritional Levels

Fasted blood samples (3 ml) were obtained from the participants through venipuncture and put into a separation gel coagulation tube and then immediately (within 2 h) transported to a local study center. The blood samples in the separation gel coagulation tubes were centrifuged at 3,500 × *g*/min for 3 min at room temperature (~21°C), and then the serum was separated for measurements of the zinc, ferritin, folate, VB12, 25-OH VD, and retinol (vitamin A: VA) concentrations. Serum zinc levels were detected by flame atomic absorption spectroscopy (AA6300 C atomic absorption spectrophotometer, Shimadzu, Japan) at 248.3 nm using hollow cathode lamps (Perkin-Elmer). The ferritin, folate, and VB12 levels were measured by chemiluminescence microparticle immunoassay (CMIA) kits (Abbott Ireland Diagnostics Division, Longford, Ireland). Serum concentrations of VD were estimated by an immunoassay method (Abbott, 3L52, USA, and Abbott i2000SR, USA). The quantitative determination of serum VA levels was performed by high-performance liquid chromatography (HPLC) according to previously described methods with an HPLC instrument (DGU-20As; C18; 315 nm; Shimadzu, Japan) (18). Of the sample from Hainan province, the serum was separated and stored at −80 immediately and transferred to Chongqing by cold-chain transportation within 2 days. All biochemical assessments of micronutrients were performed by inspectors at the pediatric laboratory of Chongqing Medical University, China.

The manufacturer-defined deficiency level was below 9.2 μmol/L for zinc, below 4.63 ng/ml for females and 21.8 ng/ml for males for ferritin, below 3.1 ng/ml for folate, and below 87 pg/ml for VB12 (11). According to the global consensus recommendations, the clinical cutoff values for VD were defined as follows: adequate ≥20 ng/ml, inadequate ≥12–20 ng/ml, and deficient <12 ng/ml (24). Besides, according to the WHO criteria, serum retinol concentrations >1.05, 0.7–1.05, and <0.7 μmol/L were defined as VA normal (VAN), marginal VA deficiency (MVAD), and VA deficiency (VAD), respectively (25).

### Statistical Analysis

The data were analyzed using the SPSS statistical software (version 19.0, SPSS Inc., USA) (26). The normality test of each dataset was performed by the Kolmogorov–Smirnov goodness-of-fit test. Continuous variables are presented as the means (with SD) or medians (with interquartile ranges). Categorical variables are described as percentages. The one-way ANOVA with the Bonferroni *post hoc* test, chi-square test, and Fisher's exact test were used to compare variables among groups. Non-parametric statistical analysis was performed with the Kruskal–Wallis H test for abnormal continuous variable comparisons. *P*-value was adjusted in multiple comparisons by the Bonferroni *post hoc* test. Associations for micronutrients with symptoms and development levels were assessed with partial correlation analysis. For all analyses, *P* ≤ 0.05 was recognized as significant.

## Results

### Characteristics of Participants

There were 738 ASD children and 302 TD children recruited between August 2016 and October 2018. They were assigned to four groups: ASD children in Chongqing (379 boys and 66 girls, aged 4.39 ± 1.25 years), TD children in Chongqing (102 boys and 99 girls, aged 4.44 ± 0.82 years), ASD children in Hainan province (250 boys and 43 girls, aged 4.28 ± 1.37 years), and TD children in Hainan province (50 boys and 51 girls, aged 4.35 ± 1.18 years). The clinical characteristics of the children are shown in Table 1. There were no significant differences in family economic status and parental education levels among the four groups of children. ASD children were more likely to be reported as with severe picky eating in both regions (29.89% in ASD children vs. 8.96% in TD children of Chongqing, adjusted *P* < 0.001; 25.6% in ASD children vs. 6.93% in TD children of Hainan, adjusted *P* < 0.001). ASD children were reported to have a higher prevalence of gastrointestinal problems in both regions (57.08% in ASD children vs. 25.87% in TD children of Chongqing, adjusted *P* < 0.001; 54.95% in ASD children vs. 20.79% in TD children of Hainan, adjusted *P* < 0.001). There was no statistical difference at prevalence of picky eating or gastrointestinal problems in ASD children of the two regions.

**Table 1:** Demographic characteristics of autistic and typically developing children in Chongqing and Hainan.

**Characteristics**		**ASD-Chongqing**		**TD-Chongqing**		**ASD-Hainan**		**TD-Hainan**		***P***
**Age (years)**	Mean ± SD	4.39 ± 1.25		4.44 ± 0.82		4.28 ± 1.37		4.35 ± 1.18		0.469
**Sex**	Male, % (*n*)	85.17	(379)	50.75	(102)	85.32	(250)	49.5	(50)	
**Ethnicity**	Han, % (*n*)	97.08	(432)	96.02	(193)	91.81	(269)	96.04	(97)	
**Family annual income per capita (RMB yuan), %(*****n*****)**										
<30,000		64.49	(287)	62.19	(125)	55.29	(162)	56.44	(57)	0.065
>30,000		35.51	(158)	37.81	(76)	44.71	(131)	43.56	(44)	
**Father's educational levels, %(*****n*****)**										
High school or below		55.06	(245)	53.23	(107)	47.1	(138)	45.54	(46)	0.102
College or above		44.94	(200)	46.77	(94)	52.9	(155)	54.46	(55)	
**Mother's educational levels, %(*****n*****)**										
High school or below		65.39	(291)	56.72	(114)	62.12	(182)	55.45	(56)	0.095
College or above		34.61	(154)	43.28	(87)	37.88	(111)	44.55	(45)	
Mild picky eating, %(*n*)		42.47	(189)	45.27	(91)	38.57	(113)	33.66	(34)	0.181
Severe picky eating, %(*n*)		29.89	(133)	8.96	(18)^a^^***^	25.6	(75)	6.93	(7)^b^^***^	<0.001
Gastrointestinal problems, %(*n*)		57.08	(254)	25.87	(52)^a^^***^	54.95	(161)	20.79	(21)^b^^***^	<0.001

*Data shown as (mean ± SD) or percentage (number). One-way ANOVA and chi-square test were used in the analysis*.

a *Significant difference of percentage of severe picky eating and gastrointestinal problems between ASD children and TD children in Chongqing*.

b *Significant difference of percentage of severe picky eating and gastrointestinal problems between ASD children and TD children in Hainan province. P-value was adjusted in multiple comparisons by the Bonferroni method*.

*** *P < 0.001*.

*ASD, autism spectrum disorder; TD, typically developing*.

### Comparison of Symptom Scores in ASD Children Between Chongqing and Hainan Province

**Figure 1:**
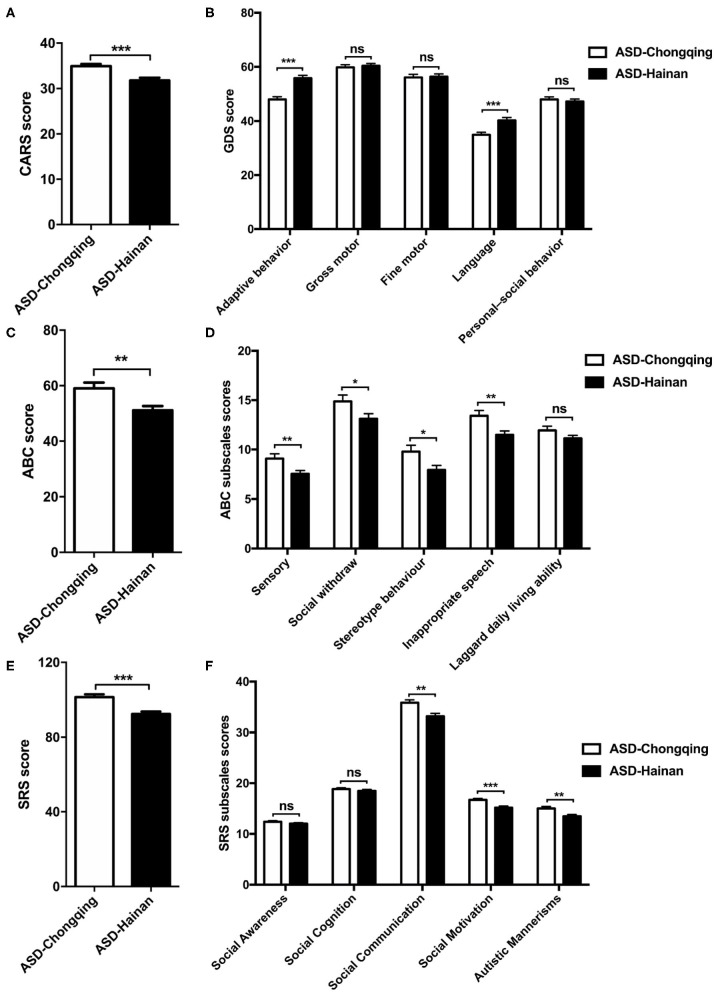
Comparison of CARS, GDS, ABC, and SRS scores in children with autism in Chongqing and Hainan. The CARS **(A)**, GDS **(B)**, ABC **(C)**, SRS **(E)** scores, subscales scores of ABC **(D)**, and SRS **(F)** of ASD children in Chongqing (*n* = 445) and Hainan (*n* = 293) were compared. The values are the means ± SEMs. The *t*-test test was used for the comparison; *P < 0.05, **P < 0.01, ***P < 0.001.

The mean CARS score, SRS score, and ABC score of the ASD children in Chongqing were significantly higher compared to those in Hainan province (*P* < 0.05, respectively, shown in Table Supplement 1; Figures 1A,C,E). Regarding the ABC subscales, significant differences were found at sensory, social withdrawal, stereotypic behavior, and inappropriate speech, and there is no significant difference at laggard daily living ability (Figure 1D). At the SRS subscales of the social motivation, social communication, and autistic mannerisms, the ASD children in Chongqing had higher scores than those in Hainan province (Figure 1F). When comparing the score of GDS, the autistic children in Chongqing had lower scores at adaptive behavior and language than those in Hainan province (Figure 1B). The data above indicate that the ASD children recruited from Chongqing have severer autistic symptoms and poorer cognitional and behavioral development than those in Hainan province.

### Anthropometry

Table 2 summarizes the Z-scores of all participants. No significant differences at the mean ZWA, ZHA, and ZBMIA were observed among the four groups. The mean ZHCA of ASD children were lower than those of local TD children, both in Chongqing and Hainan province (adjusted *P* = 0.006, 0.009, respectively). Further, the percentages of short stature, wasting, and overweight had no significant differences among the four groups. When compared with local TD children, ASD children had higher percentage of undersized head circumference in both regions (10.34 vs. 3.48% in Chongqing, adjusted *P* = 0.009; 8.19 vs. 1.98% in Hainan, adjusted *P* = 0.03). ASD children had a higher percentage of oversized head circumference in Chongqing (6.52 vs. 1%, adjusted *P* = 0.006).

**Table 2:** Growth assessment of autistic and typically developing children in Chongqing and Hainan.

**Growth Assessment**	**ASD-Chongqing**		**TD-Chongqing**		**ASD-Hainan**		**TD-Hainan**		***P***
Z_HA_, mean ± SD	−0.1 ± 1.28		−0.07 ± 1.1		−0.17 ± 1.33		−0.23 ± 0.98		0.680
Z_WA_, mean ± SD	0.14 ± 1.27		0.22 ± 1.18		0.11 ± 1.33		0.15 ± 1.03		0.834
Z_BMIA_, mean ± SD	0.35 ± 1.25		0.48 ± 1.08		0.45 ± 1.16		0.2 ± 1.11		0.202
Z_HCA_, mean ± SD	−0.2 ± 1.51		0.21 ± 1.06^a^^**^		−0.25 ± 1.07		−0.13 ± 0.94^b^^**^		<0.001
Short stature (Z_HA_ < −2), %(*n*)	7.64	(34)	5.97	(12)	2.39	(10)	2.97	(3)	0.059
Wasting (Z_BMIA_ < −2), %(*n*)	1.12	(5)	1	(2)	0	(0)	0	(0)	0.223^a^
Overweight (Z_BMIA_ > 2), %(*n*)	11.01	(49)	11.44	(23)	6.14	(18)	6.93	(7)	0.078
Undersized head circumference (ZHCA < −2), %(*n*)	10.34	(46)	3.48	(7)^a^^**^	8.19	(24)	1.98	(2)^b^^*^	0.002
Oversized head circumference (ZHCA > 2), %(*n*)	6.52	(29)	1	(2)^a^^**^	3.41	(10)	0.99	(1)	0.002

*Data shown as (mean ± SD) or percentage (number). One-way ANOVA, chi-square test, and Fisher's exact test were used in the analysis*.

a *Significant difference of Z_HCA_ and percentage of abnormal head circumference size between ASD children and TD children in Chongqing*.

b *Significant difference of Z_HCA_ between ASD children and TD children in Hainan. P-value was adjusted in multiple comparisons by the Bonferroni method*.

* *P < 0.05*,

** *P < 0.01*.

*ASD, autism spectrum disorder, TD, typically developing*.

### Diet of Children With ASD in the Two Regions

A total of 611 ASD children (367 in Chongqing, 244 in Hainan) and 289 TD children (193 in Chongqing, 96 in Hainan) completed the FFQ survey. Surveys with incomplete answers or suspected unreal answers were excluded. Table 3 shows the mean intake of food groups (g/day) in children between the two regions.

**Table 3:** Mean intake of food groups (g/day) in ASD children in Chongqing and Hainan.

**Food groups**	**ASD-Chongqing**	**TD-Chongqing**	**ASD-Hainan**	**TD-Hainan**	**χ2**	***P***
Grains	99.7 (92.9–149.5)	75 (48.5–100)	75 (10.7–100)^c^^**^	75 (39.3–100)^b^^*^	30.472	<0.001
Whole grains	2.9 (0–5.7)	10.7 (1.8–28.6)^a^^***^	2.9 (0.7–5.7)	20 (8.6–26.8)^b^^***^	83.645	<0.001
Red meat	50 (28.5–99.7)	49.8 (21.5–100)	50 (28.6–75)	50 (28.5–74.1)	9.058	0.029
Poultry meat	7.1 (0–14.3)	14.3 (1.7–28.7)^a^^***^	50 (28.6–75)^c^^***^	50 (21.4–75)	259.865	<0.001
Freshwater fish	3.3 (0–7.1)	7.1 (3.3–14.3)^a^^***^	0 (0–7.1)	1.8 (0–14.3)	28.861	<0.001
Seafood	0 (0–1.8)	7.1 (1.7–10.7)^a^^***^	21.4 (5–35.7)^c^^***^	21.4 (7.1–48.6)	283.9	<0.001
Milk and dairy products	139.5 (40–150.5)	140 (98.8–279.1)^a^^*^	140 (40–280)^c^^*^	170 (100–280)^b^^**^	11.466	0.009
Eggs	28.6 (7.1–49.8)	35.7 (16.6–50)	28.6 (14.3–50)	33 (17.9–50)	5.782	0.123
Beans and soy products	1.4 (0.6–4.3)	8.6 (2.9–11.4)^a^^***^	1.4 (0–2.9)^c^^***^	8.5 (1.2–10)^b^^**^	135.606	<0.001
Vegetables	85.7 (42.5–149.5)	111.4 (51.4–179.8)^a^^***^	78.6 (35.7–114.3)	111.6 (50–162.5)^b^^**^	23.788	<0.001
Fruits	74.8 (21.4–103.1)	74.8 (32.4–123.2)^a^^*^	75 (51.6–117.9)^c^^***^	107.1 (54.2–158.7)^b^^*^	30.217	<0.001

*Data shown as median (25–75%). The Kruskal–Wallis H test was performed for abnormal continuous variable comparisons. P-value was adjusted in multiple comparisons by Bonferroni method*.

a *Significant difference between autism children compared with typically developing children in Chongqing*.

b *Significant difference of autism children compared with typically developing children in Hainan province*.

c *Significant difference of autism children in Chongqing compared with autism children in Hainan province*.

* *P < 0.05*,

** *P < 0.01*,

*** *P < 0.001*.

*ASD, autism spectrum disorder; TD, typically developing*.

Compared with local TD children, ASD children consumed fewer whole grains, milk and dairy products, beans and soy products, vegetables, and fruits in both regions (*P* < 0.05, respectively). The grain and meat consumption of ASD children in both regions can meet the Chinese Dietary Guidelines (2016) for Chinese preschoolers by the Chinese Nutrition Society (27); however, the consumption of egg, milk and dairy products, beans and soy products, vegetables, and fruits has not met the recommendations in both regions.

When comparing the food intake of the ASD children in Chongqing with those in Hainan, the ASD children in Chongqing consumed fewer poultry meat, seafood, milk and dairy products, and fruits compared with those in the ASD children in Hainan, while there was more grain, bean, and soy product consumption.

Table Supplement 2 shows the food groups selectivity of ASD and TD children in two regions. Compared with local TD children, ASD children had higher rates of omitting eggs, milk and dairy products, and fruits in both regions. When comparing food group selectivity of the ASD children in Chongqing with those in Hainan, the ASD children in Chongqing had higher rates of omitting whole grains, seafood, and fruits, and lower rates of omitting grain, freshwater fish, beans, and soy products (*P* < 0.05, respectively).

### Biochemical Determination for Nutritional Levels

**Figure 2:**
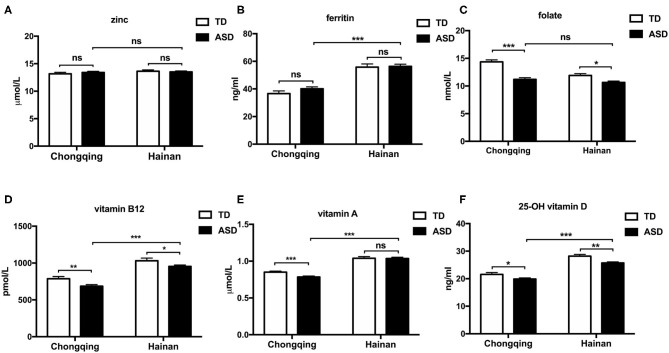
Serum micronutrient levels of ASD and typically developing children in Chongqing and Hainan. Children were assigned to four groups: ASD-Chongqing (*n* = 445), TD-Chongqing (*n* = 201), ASD-Hainan (*n* = 293), and TD-Hainan (*n* = 101). The serum concentrations of zinc **(A)**, ferritin **(B)**, folate **(C)**, vitamin B12 **(D)**, vitamin D **(F)**, and vitamin A **(E)** of children were detected and compared. The values are the means ± SEMs. The one-way ANOVA test was used for the comparison, *P-*value was adjusted in multiple comparisons by the Bonferroni method. *P < 0.05, **P < 0.01, ***P < 0.001. ASD, autism spectrum disorder; TD, typically developing.

Figure 2 shows the comparison of the serum biochemical indices for micronutrient levels. In Chongqing, the mean serum VA, folate, VB12, and VD levels in ASD children were significantly reduced compared to those in local TD children (*P* < 0.05, respectively). The mean zinc and ferritin levels between the ASD children and local TD children in Chongqing were not statistically different. In Hainan province, ASD children had significantly reduced levels of serum folate, VB12, and VD than local TD children (*P* < 0.05, respectively). The mean zinc, ferritin, and VA levels between the ASD children and local TD children in Hainan were not statistically different. Comparing ASD children with local TD children, folate, VB12, and VD were consistently lower in both Chongqing and Hainan province.

When comparing the ASD children in Chongqing with those in Hainan province, the mean serum ferritin, VA, VB12, and VD concentrations were lower in the ASD children in Chongqing. Therefore, it seemed that the ASD children in Hainan province have greater micronutrient status than those in Chongqing.

### Micronutrient Deficiency Rates in ASD and TD Children

We compared the micronutrient deficiency rates of all groups, shown in Table 4.

**Table 4:** Micronutrient deficiency rates in ASD and typically developing children in Chongqing and Hainan.

**Micronutrients**		**ASD-Chongqing**	**TD-Chongqing**	**ASD-Hainan**	**TD-Hainan**	***P***
Zinc		7.64 (34)	0.5 (1)^a^^***^	1 (3)^c^^***^	0 (0)	<0.001
Ferritin		15.7 (70)	16.9 (34)	4.1 (12)^c^^***^	2 (2)	<0.001
Folate		0.2 (1)	0 (0)	0.3 (1)	0 (0)	1.000
Vitamin B12		0 (0)	0 (0)	0 (0)	0 (0)	
Vitamin D	deficiency	8.8 (39)	4 (8)^a^^*^	1.7 (5)^c^^***^	1 (1)	<0.001
	inadequacy	49.7 (221)	59.4 (119)^a^^*^	18.4 (54)	2 (2)^b^^***^	
Vitamin A	deficiency	38.4 (171)	21.4 (43)^a^^***^	13.7 (40)^c^^***^	7.9 (8)	<0.001
	marginal deficiency	46.7 (208)	41.3 (83)	36.9 (108)^c^^*^	45.5 (46)	

*Data shown as percentage (number). Chi-square test, and Fisher's exact test were used in the analysis*.

a *Significant difference of percentage of micronutrient deficiency of autism children compared with typically developing children in Chongqing*.

b *Significant difference of percentage of micronutrient deficiency of autism children compared with typically developing children in Hainan province*.

c *Significant difference of percentage of micronutrient deficiency of autism children in Chongqing compared with autism children in Hainan province*.

*P-value was adjusted in multiple comparisons by the Bonferroni method*.

* *P < 0.05*,

*** *P < 0.001*.

*ASD, autism spectrum disorder; TD, typically developing*.

When compared with the local TD children, the ASD children in Chongqing had higher deficiency rates of zinc, VD, and VA, while there were no significant differences in ferritin, folate, and VB12. The ASD children in Hainan had higher inadequacy rate of VD than the local TD children, but they did not differ in deficiency rates of all the tested micronutrients.

There are no significant differences between ASD and TD children in Hainan province in deficiency rates of all tested micronutrients.

When comparing the deficiency rates of micronutrients of the ASD children in the two regions, the ASD children in Chongqing had higher deficiency rates of zinc, ferritin, VA, and VD than those of the ASD children in Hainan. These results suggest that autistic children in Hainan province have greater micronutrient status than those in Chongqing.

The greatest deficiency in all groups was observed for VA (7.92–38.43%), followed by ferritin (1.98–16.92%). Moreover, the marginal VA deficiency rate was considerable in children of all groups, and inadequacy of VD cannot be ignored either.

### Correlation Analysis of the Micronutrient Level for ABC, SRS, CARS, and DQ Scores of Autistic Children

Associations for micronutrients with symptoms and development levels were assessed with partial correlation analysis (Table 5). According to this analysis, there was a negative association between the serum level of VA with ABC (*r* = −0.211, *P* = 0.009) and SRS scores (*r* = −0.186, *P* = 0.013), and also a negative association between VD levels and SRS scores (*r* = −0.122, *P* = 0.037), while folate levels revealed a negative association with CARS scores (*r* = −0.228, *P* < 0.001). These results suggest that VA, VD, and folate may be related to symptoms of ASD children. VD (*r* = 0.24, *P* < 0.001) and zinc (*r* = 0.113, *P* = 0.048) levels revealed a positive association with DQ scores. This finding suggests that VD and zinc may be protective factors of neurodevelopment in ASD children. There were no statistical correlations between VB12 and ferritin with clinical measure scores.

**Table 5:** Partial correlation analysis of the micronutrient level for ABC, SRS, CARS, and DQ scores of autistic children.

**Variables**	**ABC**		**SRS**		**CARS**		**DQ**	
	***r***	***P***	***r***	***P***	***r***	***P***	***r***	***P***
Vitamin A	−0.211	0.009	−0.186	0.013	−0.13	0.066	0.1	0.329
Vitamin D	−0.057	0.124	−0.122	0.037	−0.142	0.059	0.24	<0.001
Ferritin	−0.03	0.476	−0.069	0.245	−0.018	0.717	0.147	0.102
Vitamin B12	−0.04	0.396	−0.057	0.212	−0.072	0.142	0.037	0.461
Folate	0.031	0.510	0.044	0.336	−0.228	<0.001	0.088	0.064
Zn	0.026	0.537	0.077	0.055	0.069	0.121	0.113	0.048

## Discussion

In the cross-sectional research, we evaluated the nutritional status of the ASD children in Chongqing and Hainan province, and compared the ASD symptoms between the two different regions, to explore the correlations between the nutritional status and ASD symptoms.

The anthropometry parameter is a vital indicator of an individual's nutritional status. Findings on the anthropometric measurement of ASD children were inconsistent. Some studies showed that children with ASD likely have a higher risk of overweight and obesity (28–30) compared with children without autism, although some studies did not find the difference (31). The inconsistent anthropometric findings could be ascribed to different genetic backgrounds, diet cultural patterns, and economic levels. Sun et al. found that the mean BMI, ZWH, and ZBMIA of the ASD children were higher than those of the TD children in the northeast of China (32). However, no significant differences in the ZWA, ZHA, and ZBMIA were observed between the ASD and the TD children in the two regions of our study. In addition, an increased prevalence of overweight was not shown in ASD children. Our results also corroborated the research suggesting that the eating behavior of ASD is often a problem of dietary variety, not inadequate or excessive volume, and they are not significantly different from neurotypical children on anthropometric measurements.

Multiple studies showed abnormal growth of head circumference and brain size in early infancy of ASD children. A meta-analysis showed that the proportion of macrocephaly in ASD children was largely more than that of the controls (15.7 vs. 3%, respectively) (33). The study of Libero et al. suggested that there may be a subgroup of ASD with disproportionate brain-to-body size in early childhood (34). Suren et al. also found parallel head growth in autistic boys compared with controls, while the autism group had higher variability and prevalence of macrocephaly (35). Our results also showed that the mean head size of ASD children was below than that of local TD children, and ASD children had a higher percentage of abnormal head circumference than TD children. Abnormal brain growth in autism can seemingly stem from several different mechanisms, it is still not entirely clear, and abnormal head circumference may be a prospective indicator to predict the diagnosis of autism (36).

Feeding problems, such as food selectivity, food refusal, and abnormal dietary patterns, are highly prevalent in ASD children (4, 37–41). These dietary problems could cause the development of nutrient deficiency and consequently worsen autistic symptoms. ASD children often prefer snacks, starches, and processed foods, and reject vegetables, fruits, and proteins (4, 37, 39). In our study, we found that ASD children had increased rates of food selectivity, and they consumed fewer whole grains, milk and dairy products, beans and soy products, vegetables, and fruits than local TD children in both regions. When comparing the food intake of ASD children in Chongqing with those in Hainan, the ASD children in Hainan consumed more poultry meats, seafood, milk and dairy products, and fruits compared with the ASD children of Chongqing. It seemed that the ASD children in Hainan have healthier dietary habits. However, the ASD children's consumption of egg, milk and dairy products, beans and soy products, vegetables, and fruits has not met the recommendation in both regions.

Nutrients are a vital role in neurodevelopment during early life (42–44). Malnutrition could have a remarkable adverse effect on neurodevelopment and brain function, and nutrient deficiency is closely correlated with neurodevelopmental problems in children. It is challenging to achieve adequate nutrition intake for ASD children because of their gastrointestinal problems, selective eating pattern, and metabolic abnormalities (45). Multiple studies have demonstrated the inadequacy of various nutrients in ASD children, including vitamins A, C, B6, B12, D, E, K, folate, zinc, calcium, and iron (7, 12, 29, 37, 40). In our study, when comparing autistic children with local typically developing children, folate, VB12, and VD are consistently lower in both Chongqing and Hainan province. The greatest deficiency in all groups was observed for VA, followed by ferritin and VD.

The top food sources of VA and VD are milk, fish, seafood, eggs, and fortified food, and the main natural source of VD comes from exposure to UVB in sunlight. In our study, children with ASD in both regions tend to omit most kinds of food rich in VA and VD. The ASD children in Chongqing had a higher prevalence of omitting fish and seafood than those in Hainan. What is more, Hainan is a tropical island with year-round high solar irradiance, while longer rainy seasons and higher smog levels in Chongqing lead to less solar irradiance exposure. We have reason to believe that the ASD children in Chongqing may have a higher risk of VA and VD deficiency than those in Hainan province.

The role of VD in ASD has attracted increasing interest in recent research (46–48). VD is believed to be closely related to ASD, though the potential mechanisms are not clear yet. Syed and coworkers reviewed published studies from different latitudes to show a correlation between low solar irradiance and ASD (49). A meta-analysis revealed that serum VD levels in ASD children are significantly decreased compared with neurotypical children (9).

In our previous research, the rate of VA deficiency (including marginal deficiency) in ASD children was up to 77.9%, and the VA level was inversely correlated with the CARS score (11). Further, the social interaction of ASD children was improved after VA supplements (50). Our research also proved the considerable prevalence of VA deficiency in ASD children and a negative association between the serum concentrations of VA and VD and the severity of symptoms. As a one-carbon donor, folate is essential for numerous metabolic biochemical processes. Many studies showed the association between genetic and abnormalities of folic acid and the pathogenesis of ASD (51–56). In addition, in our study, there was a negative association between folate and severity of ASD symptom. Notably, the strengths of the correlations between micronutrients and symptoms are not very high, for the causes and mechanisms of autism are complex and uncertain, and nutrition is only one of the numerous factors associated with the occurrence and progress of ASD. Micronutrients are pivotal environmental factors, and their influence on ASD should not be ignored, for malnutrition is more preventable and treatable than other factors.

The present study is not without potential limitations. Even though the two regions may be comparable in terms of economic conditions, we did not take into account bias and equivalence in measurement of the instruments (CARS, ABC, SRS, and GDS) for different local dialects and cultures (57); thus, the possibility that differences of scores of measurement might partly be attributable to bias cannot be excluded. We did not consider differences in other environmental, genetic, and biological factors between the two regions, and it may be different in the extent of factors each contributes to the progress of ASD in different areas and population. Our data do not allow for the distinction causal or confounders of the association between nutrition status and symptoms of ASD. There may be a mutual cause–effect relationship between nutrition status and symptoms of ASD. Owing to unusual eating patterns and some interior genetic and pathological factors, ASD children have a high risk of malnutrition, and nutritional status could influence the development of ASD and aggravate their symptoms in turn. Further research should consider causes, consequences, and remediation.

## Conclusion

ASD children exhibit a higher risk of nutrient deficiencies than neurotypical children, and there are regional differences in the nutritional status of ASD children. It seemed that the ASD children in Hainan province have greater micronutrient status and less severe autistic symptoms compared with those in Chongqing. Micronutrients VA, VD, folate, and zinc levels were associated with ASD symptoms and development of ASD children. Therefore, it is essential to provide detailed nutritional evaluation and individualized nutrition interventions for ASD children from different backgrounds.

## Data Availability Statement

The datasets generated for this study are available on request to the corresponding author.

## Ethics Statement

The studies involving human participants were reviewed and approved by Chinese Clinical Trial Registry (ChiCTR) (registration number: ChiCTR-ROC-14005442). Written informed consent to participate in this study was provided by the participants' legal guardian/next of kin.

## Author Contributions

JZ, JC, LL, and TL were involved in designing the research and writing the research protocol, analyzing the data, and writing the manuscript. JC, LL, and TL were also involved in supervising subjects' recruitment, data collection, and drafting the manuscript. JZ, MG, TY, XL, and TT were engaged in questionnaire surveys, data collection and analysis, and revising the paper. LL and TL had primary responsibility for the final content. All authors approved the final version of the manuscript.

### Conflict of Interest

The authors declare that the research was conducted in the absence of any commercial or financial relationships that could be construed as a potential conflict of interest.

## Abbreviations

ABC: Autism Behavior Checklist
ASD: autism spectrum disorder
CARS: Childhood Autism Rating Scale
CMIA: chemiluminescence microparticle immunoassay
DQ: developmental quotient
FFQ: food frequency questionnaire
GDS: Gesell Developmental Scale
HPLC: high-performance liquid chromatography
MTHFR: methylenetetrahydrofolate reductase
SRS: Social Responsiveness Scale
TD: typically developing
VA: vitamin A
VAD: vitamin A deficiency
MVAD: marginal vitamin A deficiency
VAN: vitamin A normal
VB12: vitamin B12
VD 25-OH: vitamin D
ZBMIA: Z-scores of body mass index-for-age
ZHA: Z-scores of height-for-age
ZHCA: Z-scores of weight-for-head circumference
ZWA: Z-scores of weight-for-age.
