# Supplementary figures and images for: Nutritional Status and Symptoms in Preschool Children With Autism Spectrum Disorder: A Two-Center Comparative Study in Chongqing and Hainan Province, China
Source: Front Pediatr. 2020 Sep 3;8:469. doi: 10.3389/fped.2020.00469 (PMC7494825; doi:10.3389/fped.2020.00469)

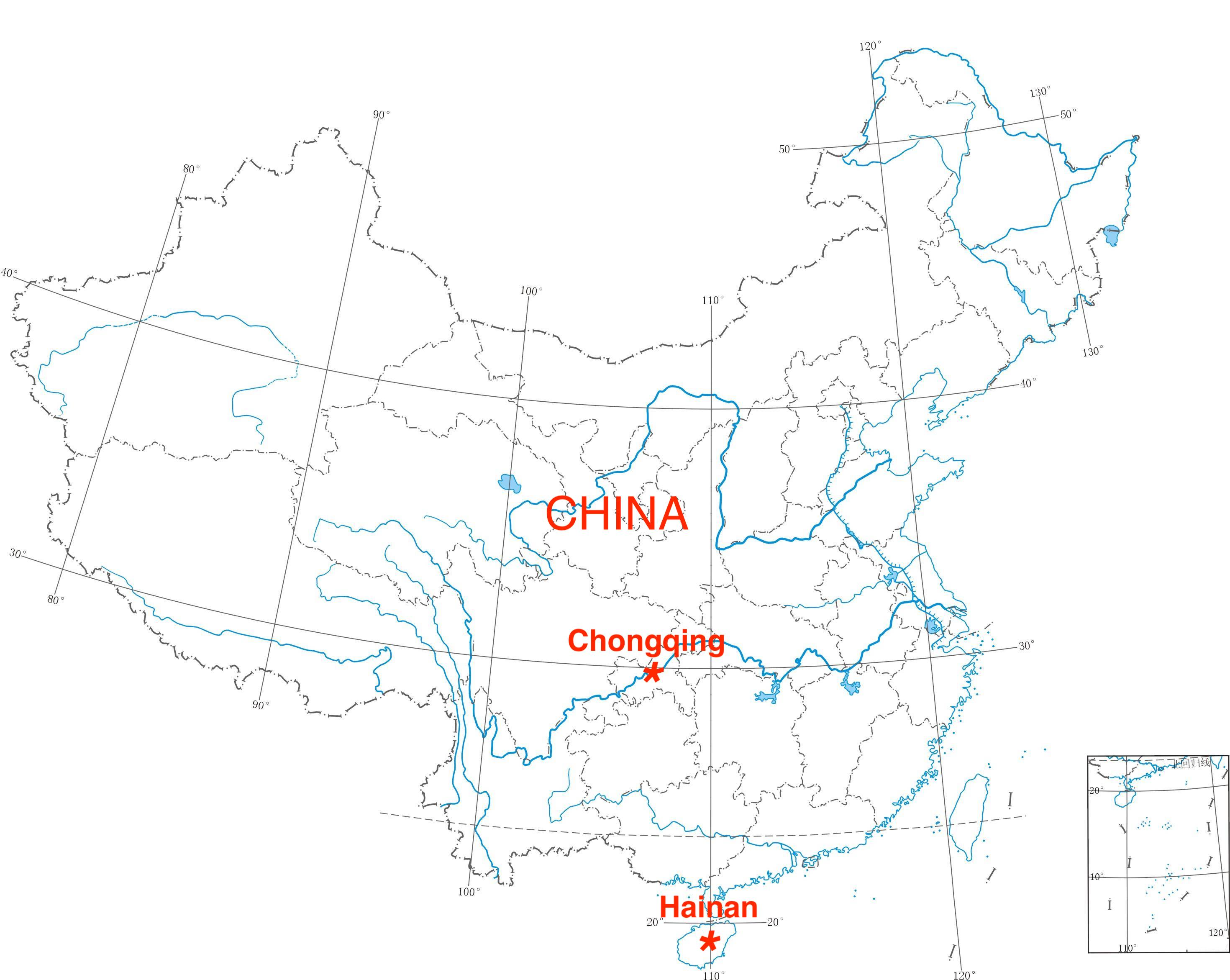

Supplement: Supplementary file 1 [file Image_1.tiff]
