# Supplementary material for: Nutritional Status and Symptoms in Preschool Children With Autism Spectrum Disorder: A Two-Center Comparative Study in Chongqing and Hainan Province, China
Source: Front Pediatr. 2020 Sep 3;8:469. doi: 10.3389/fped.2020.00469 (PMC7494825; doi:10.3389/fped.2020.00469)
Supplement: Supplementary file 2 [file Image_2.pdf]

Additional file 2-Figure supplement 2 participant flow chart.

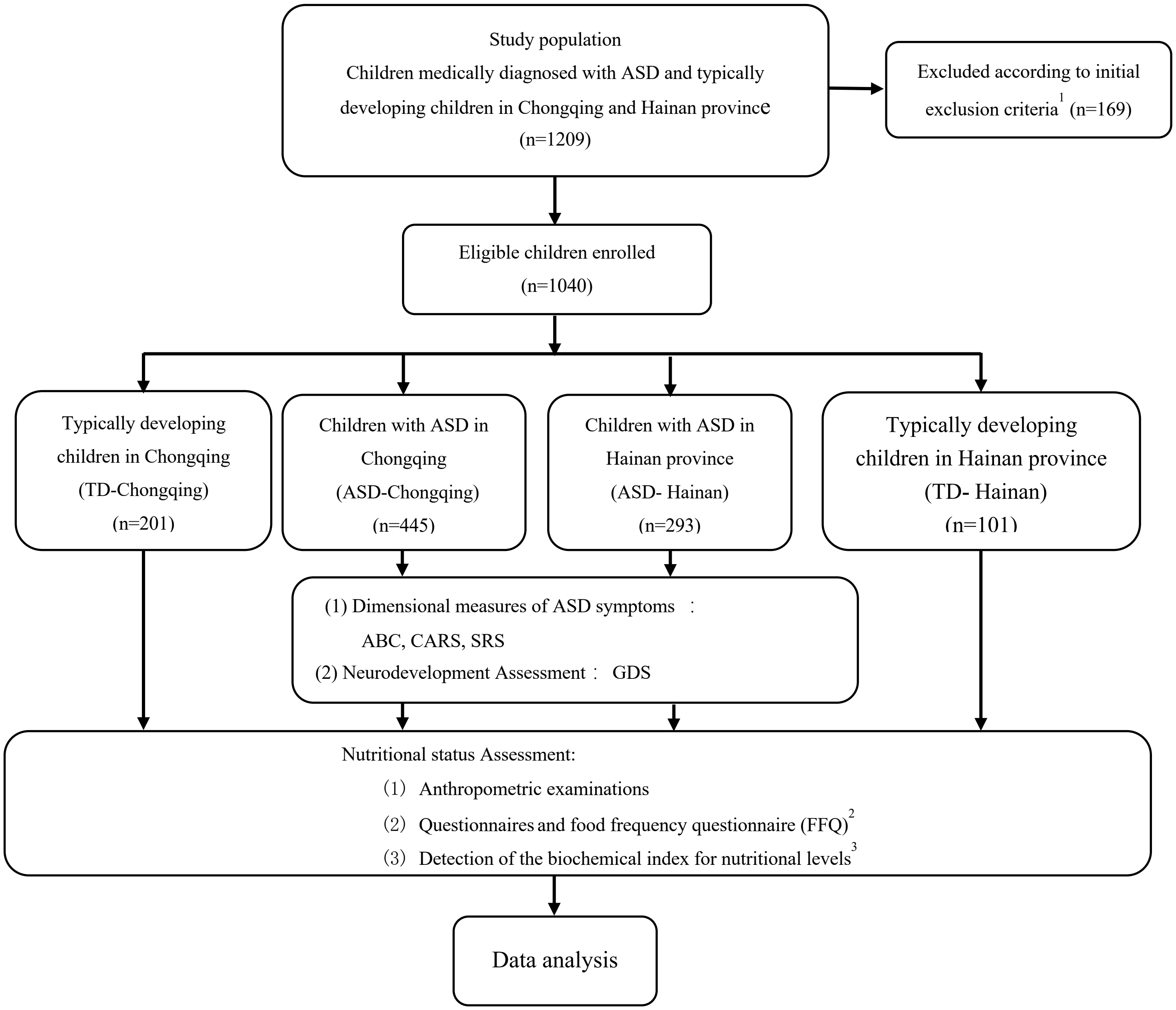

[1]The exclusion criteria included a history of other developmental disorders or psychiatric diseases, such as genetic disorders with comorbid autism, Rett Syndrome, cerebral palsy, chronic seizures, other congenital diseases, recent infection, and recent (within 6 months) use of vitamin or mineral supplementation.

[2] The following data were obtained with questionnaires from all children(n=1040): the children's general condition (e.g. name, age, gender, ethnicity, family economic status and parental education level), medical history, eating behavior, gastrointestinal problems, etc.

A total of 611 ASD children (367 in Chongqing, 244 in Hainan) and 289 TD children (193 in Chongqing, 96 in Hainan) completed the FFQ survey. Surveys with uncompleted answers or suspected unreal answers were excluded.

[3] The serum concentrations of Zinc (Zn), ferritin (FER), folate (FOL), vitamin B12 (VB12), 25-OH vitamin D (VD), and vitamin A (retinol ,VA) of all children were detected.
