# Supplementary material for: Nutritional Status and Symptoms in Preschool Children With Autism Spectrum Disorder: A Two-Center Comparative Study in Chongqing and Hainan Province, China
Source: Front Pediatr. 2020 Sep 3;8:469. doi: 10.3389/fped.2020.00469 (PMC7494825; doi:10.3389/fped.2020.00469)
Supplement: Supplementary file 3 [file Table_1.docx]

**Table S1** **Comparison of CARS, ABC，SRS and GDS scores in children with autism in Chongqing and Hainan**

|  | **ASD-Chongqing** | **ASD-Hainan** | **t** | ***P* value** |
| --- | --- | --- | --- | --- |
| **CARS** | 36.15±7.42 | 32.19±6.51 | 7.472 | <0.0001*** |
| **ABC** |  |  |  |  |
| Sensory | 8.78±4.79 | 7.536±4.35 | 2.941 | 0.0034** |
| Social withdrawal | 14.69±7.06 | 13.19±6.12 | 2.451 | 0.0145* |
| Stereotypic behavior | 9.57±5.13 | 8.26±4.51 | 2.203 | 0.0279* |
| Inappropriate speech | 13.25±6.6 | 11.83±6.09 | 2.946 | 0.0033** |
| Laggard daily living ability | 11.91±5.57 | 10.86±5.09 | 1.593 | 0.1117 |
| Total ABC scores | 58.19±26.17 | 51.68±23.37 | 3.235 | 0.0013** |
| **SRS** |  |  |  |  |
| Social awareness | 12.45±3.58 | 11.99±3.01 | 1.793 | 0.0733 |
| Social cognition | 18.98±4.52 | 18.42±4.67 | 1.627 | 0.1042 |
| Social communication | 35.87±8.68 | 33.9±9.02 | 2.966 | 0.0031** |
| Social motivation | 17.2±5.52 | 15.9±4.88 | 3.361 | 0.0008*** |
| Autistic mannerisms | 14.92±6.45 | 13.4±5.8 | 3.259 | 0.0012** |
| Total SRS scores | 99.42±23.08 | 93.61±20.58 | 3.49 | 0.0005*** |
| **GDS** |  |  |  |  |
| adaptive behavior | 47.98±17.72 | 55.74±17.67 | 5.303 | <0.0001*** |
| Gross motor | 59.8±18.25 | 60.32±14.87 | 0.3741 | 0.7084 |
| Fine motor | 56.07±19.97 | 56.31±17.11 | 0.1508 | 0.8802 |
| language | 34.93±15.96 | 40.2±17.88 | 3.777 | 0.0002*** |
| Personal-social behavior | 47.97±17.11 | 47.14±15.4 | 0.6135 | 0.5398 |

The values are the means ± SEMs. The t-test test was used for the comparison, **P* < 0.05, ***P*< 0.01, ****P* < 0.001. ASD= autism spectrum disorder, ABC=Autism Behavior Checklist, CARS=Childhood Autism Rating Scale, SRS=Social Responsiveness Scale, GDS =Gesell Developmental Scale
