# Supplementary material for: Nutritional Status and Symptoms in Preschool Children With Autism Spectrum Disorder: A Two-Center Comparative Study in Chongqing and Hainan Province, China
Source: Front Pediatr. 2020 Sep 3;8:469. doi: 10.3389/fped.2020.00469 (PMC7494825; doi:10.3389/fped.2020.00469)
Supplement: Supplementary file 4 [file Table_2.docx]

**Table S2** **Food groups selectivity in ASD and typically developing children in Chongqing and Hainan**

| **Food groups** | **ASD-Chongqing** |  | **TD-Chongqing** |  | **ASD-Hainan** |  | **TD-Hainan** |  | **χ2** | ***P*** |
| --- | --- | --- | --- | --- | --- | --- | --- | --- | --- | --- |
|  | **Omitted, %(n)** | **Consumed, %(n)** | **Omitted, %(n)** | **Consumed, %(n)** | **Omitted, %(n)** | **Consumed, %(n)** | **Omitted, %(n)** | **Consumed, %(n)** |  |  |
| Grains | 5.2(19) | 94.8(348) | 4.1(8) | 95.9(185) | 18(44) | 82(200)c | 10.4(10) | 89.6(86) | 36.647 | <0.001 |
| Whole grains | 40.9(150) | 59.1(217) | 25.9(50) | 74.1(143)a | 26.2(64) | 73.8(180)c | 22.9(22) | 77.1(74) | 24.011 | <0.001 |
| Red meats | 11.2(41) | 88.8(326) | 11.9(23) | 88.1(170) | 9.8(24) | 90.2(220) | 8.3(8) | 91.7(88) | 1.14 | 0.767 |
| Poultry meats | 34.9(128) | 65.1(239) | 28(54) | 72(139) | 27.9(68) | 72.1(176) | 17.7(17) | 82.3(79) | 11.996 | 0.007 |
| Freshwater fish | 47.4(174) | 52.6(193) | 24.4(47) | 75.6(146)a | 58.2(142) | 41.8(102)c | 52.1(50) | 47.9(46) | 52.762 | <0.001 |
| Seafood | 76.8(282) | 23.2(282) | 46.1(89) | 53.9(104)a | 22.5(55) | 77.5(189)c | 19.8(19) | 80.2(77) | 215.46 | <0.001 |
| Milk and dairy products | 19.9(73) | 80.1(294) | 11.4(22) | 88.6(171)a | 17.6(43) | 82.4(201) | 6.3(6) | 93.7(90)b | 14.443 | <0.001 |
| Eggs | 16.9(62) | 83.1(305) | 8.3(16) | 91.7(177)a | 16.4(40) | 83.6(204) | 6.3(6) | 93.7(90)b | 11.5 | 0.009 |
| Beans and soy products | 25.1(92) | 74.9(275) | 21.8(42) | 78.2(151) | 40.2(98) | 59.8(146)c | 27.1(26) | 72.9(70)b | 22.715 | <0.001 |
| Vegetables | 10.9(40) | 89.1(327) | 9.3(18) | 90.7(175) | 9.4(23) | 90.6(221) | 6.3(6) | 93.7(90) | 1.964 | 0.58 |
| Fruits | 20.2(74) | 79.8(74)a | 7.8(15) | 92.2(178)a | 11.5(28) | 88.5(216)c | 5.2(5) | 94.8(91)b | 22.946 | <0.001 |

Data shown as percentage(number) of ASD children omitted or consumed food groups. The chi-square test was used to analysis. If the frequency of intake food groups is ≤1 times/month, it would be considered as “omitted” that food group in this study, if not, it would be considered as consumed that food group. ASD= autism spectrum disorder, TD= typically developing. ^a^ Significant deference between autism children compared with typically developing children in Chongqing. ^b^ Significant deference between autism children compared with typically developing children in Hainan province. ^c^ Significant deference between autism children in Chongqing compared autism children in Hainan province. *P* value was adjusted in multiple comparisons by Bonferroni method.
